# Machine learning in renal pathology

**DOI:** 10.3389/fneph.2022.1007002

**Published:** 2022-11-29

**Authors:** Matthew Nicholas Basso, Moumita Barua, Julien Meyer, Rohan John, April Khademi

**Affiliations:** ^1^ Image Analysis in Medicine Lab (IAMLAB), Department of Electrical, Computer, and Biomedical Engineering, Ryerson University, Toronto, ON, Canada; ^2^ Division of Nephrology, University Health Network, Toronto, ON, Canada; ^3^ Toronto General Hospital Research Institute, Toronto General Hospital, Toronto, ON, Canada; ^4^ Department of Medicine, University of Toronto, Toronto, ON, Canada; ^5^ Institute of Medical Sciences, University of Toronto, Toronto, ON, Canada; ^6^ School of Health Services Management, Ryerson University, Toronto, ON, Canada; ^7^ Department of Pathology, University Health Network, Toronto, ON, Canada; ^8^ Keenan Research Center for Biomedical Science, St. Michael’s Hospital, Unity Health Network, Toronto, ON, Canada; ^9^ Institute for Biomedical Engineering, Science, and Technology (iBEST), a partnership between St. Michael’s Hospital and Ryerson University, Toronto, ON, Canada

**Keywords:** digital image analysis, computational pathology, machine learning, deep learning, glomerulus, minimal change disease, membranous nephropathy, thin-basement membrane nephropathy

## Abstract

**Introduction:**

When assessing kidney biopsies, pathologists use light microscopy, immunofluorescence, and electron microscopy to describe and diagnose glomerular lesions and diseases. These methods can be laborious, costly, fraught with inter-observer variability, and can have delays in turn-around time. Thus, computational approaches can be designed as screening and/or diagnostic tools, potentially relieving pathologist time, healthcare resources, while also having the ability to identify novel biomarkers, including subvisual features.

**Methods:**

Here, we implement our recently published biomarker feature extraction (BFE) model along with 3 pre-trained deep learning models (VGG16, VGG19, and InceptionV3) to diagnose 3 glomerular diseases using PAS-stained digital pathology images alone. The BFE model extracts a panel of 233 explainable features related to underlying pathology, which are subsequently narrowed down to 10 morphological and microstructural texture features for classification with a linear discriminant analysis machine learning classifier. 45 patient renal biopsies (371 glomeruli) from minimal change disease (MCD), membranous nephropathy (MN), and thin-basement membrane nephropathy (TBMN) were split into training/validation and held out sets. For the 3 deep learningmodels, data augmentation and Grad-CAM were used for better performance and interpretability.

**Results:**

The BFE model showed glomerular validation accuracy of 67.6% and testing accuracy of 76.8%. All deep learning approaches had higher validation accuracies (most for VGG16 at 78.5%) but lower testing accuracies. The highest testing accuracy at the glomerular level was VGG16 at 71.9%, while at the patient-level was InceptionV3 at 73.3%.

**Discussion:**

The results highlight the potential of both traditional machine learning and deep learning-based approaches for kidney biopsy evaluation.

## 1 Introduction

Kidney biopsies are examined by renal pathologists using light microscopy (LM), immunofluorescence (IF), and electron microscopy (EM), to diagnose often descriptively, from the range of glomerular diseases. Digitization of whole-slide images (WSIs) has allowed for the application of software tools, from classic image analysis to more recent deep learning methods, to facilitate computer-based assistance to biopsy diagnosis. Besides increasing reproducibility and likely improving diagnostic accuracy, computer derived tools may uncover novel subvisual features and identify objective diagnostic and prognostic biomarkers.

There are limited works for automated renal pathology analysis with a few approaches in the literature that apply machine learning to the study of glomerular diseases. These have mostly included distinguishing the types of lesions that are known to occur in the glomerulus, and well as separating the degree of glomerular lesions within a specific disease. For example, Eiichiro et al. (1) constructed a deep learning model to classify seven different glomerular pathological findings, and this was found to improve diagnostic accuracy. Ginley et al. (2) used traditional image analysis and modern machine learning techniques to grade the severity of diabetic glomerular changes, and this correlated with disease severity. Other studies (3, 4) have used semantic segmentation using convolutional neural networks (CNNs) to detect and classify glomeruli as normal and sclerosed from WSIs. Taken together, this demonstrates that machine learning tools are feasible for some aspects of renal pathology classification.

Our focus in this paper is to apply machine learning techniques to automatically classify glomerular diseases from images of Periodic Acid Schiff (PAS) stained renal biopsies alone. We choose three common glomerular diseases seen clinically - minimal change disease (MCD), membranous nephropathy (MN), and thin-basement membrane nephropathy (TBMN). Although these diseases are complex in overall pathophysiology, they are distinguished by somewhat straightforward histologic principles. MCD appears normal by LM (with diffuse effacement by EM), MN shows varying degrees of capillary wall thickening by LM (with immune complexes on IF/EM), while TBMN may show some capillary wall wrinkling on LM (with definitive thinning only on EM). Thus, software tools could potentially be applied to PAS stained WSIs to detect this imaging features and diagnose disease in the absence of IF/EM. Not only does that reduce dependence on secondary modalities, equipment and expertise, but such tools could be used to increase objectivity, efficiency and understanding of disease mechanisms.

In this work, we implement and evaluate two types of machine learning frameworks for classification of MCD, MN and TBMN diseases. One method is based on CNNs which while having increased discriminatory abilities have not been applied on these glomerular diseases before. For this we develop and fine-tune three pre-trained deep learning models with data augmentation and Grad-CAM for better performance and interpretability. The other method is based on a recently published (5) biomarker feature extraction model (BFE) and a traditional machine learning classifier that uses 233 explainable features for glomerular and patient-level classification. Our study is a starting point in glomerular disease diagnosis by machine learning and encourages the field to undertake more and larger studies with regards to kidney disease diagnosis. While machine learning applied to renal pathology is still in its infancy and more work is required before automated tools can be used to supplement workflows, this paper provides the basis and groundwork for such research.

## 2 Methods

### 2.1 Data

**Figure 1:**
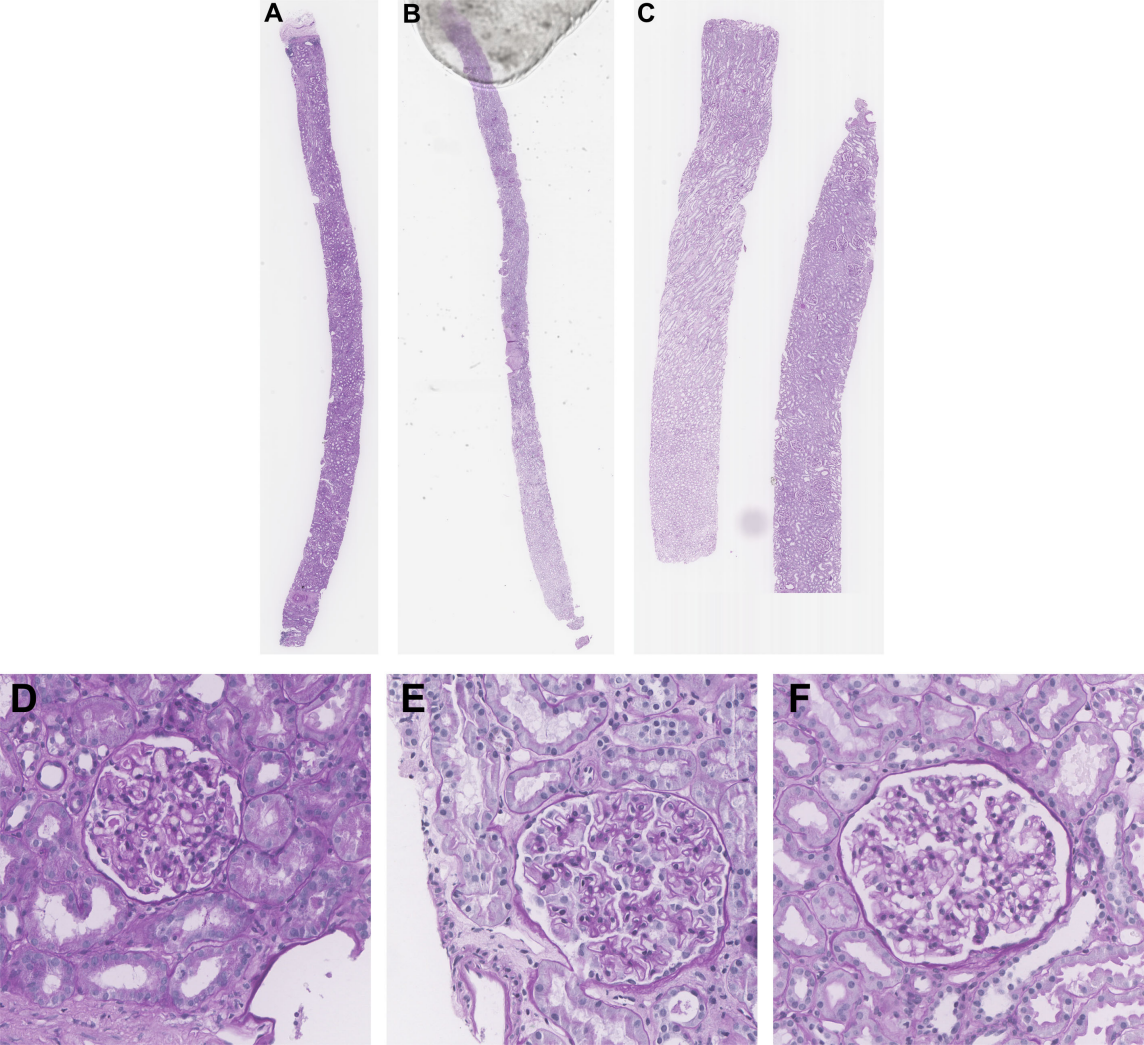
Sample kidney needle biopsies from TGH dataset and sample respective glomeruli images. **(A, D)** MCD, **(B, E)** MN, and **(C, F)** TBMN biopsy and glomerular images.

Renal biopsies were obtained from Toronto General Hospital (TGH) in Toronto, Ontario, Canada and institutional research board approval was obtained (19-5268). The dataset consists of WSIs of renal biopsies from *n* = 45 different patients with *n =* 15 WSIs per disease (MCD, TBMN, and MN), derived from pathologist (R.J.) assessment using LM, IF, and EM. Biopsies were chosen from cases with classic disease features and without changes associated with other glomerular disease. One slide from each case stained by the standard PAS method was used for analysis. Slides were scanned at 40x magnification with a resolution of 0.2526 x 0.2526 µm. Over all slides, a total of 375 glomeruli were extracted by manually cropping regions of interest (ROIs) of size 1500 x 1500 containing glomeruli and glomerular boundary segmentations were completed using Pathcore’s Sedeen Image Viewer (6). All glomeruli fully encapsulated by Bowman capsule were included. The dataset also had a total of 150 glomerular structure annotations which were previously used to validate the performance of the glomerular structure segmentation algorithm for the biomarker feature extraction model. Annotations were performed by a trained biomedical student (M.N. Basso) and validated by a pathologist (R.J.) for quality control. **Figure 1** illustrates sample WSI needle biopsies stained with standard Periodic Acid Schiff (PAS) and sample glomeruli from each WSI. **Table S1** details additional clinical information such as age, sex, and disease specific information.

### 2.2 Glomerular and patient-level classification models

**Figure 2:**
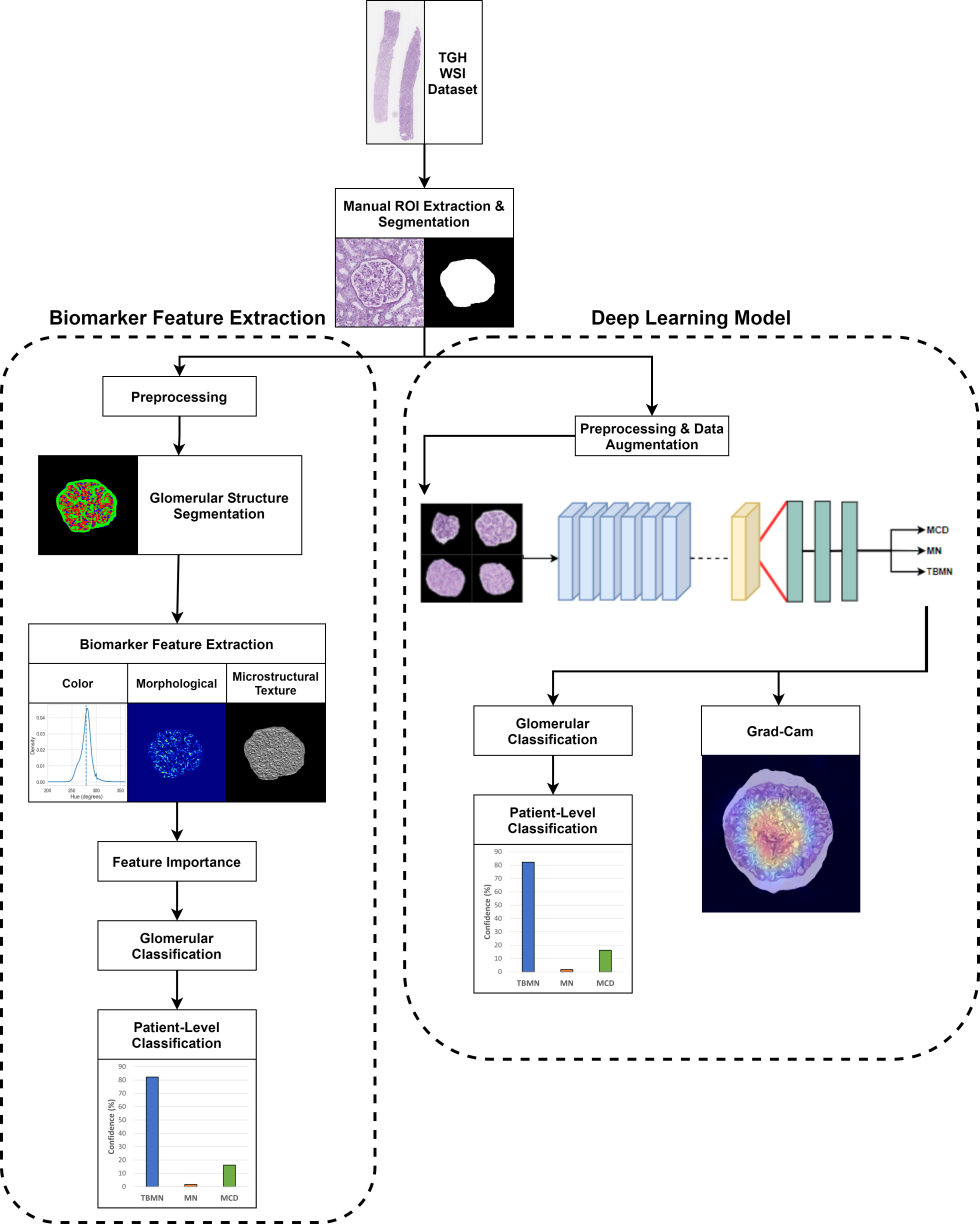
Overview of experimental design.

The biomarker feature extraction and deep learning-based computer-aided diagnostic systems were applied to light microscopy PAS images for MCD, MN, and TBMN glomerular and patient-level classification. The experimental design is shown in **Figure 2**. Both approaches incorporate techniques for model interpretability.

The biomarker feature extraction system employs image analysis techniques to extract 233 explainable biomarkers related to color, morphological, and microstructural texture features from the glomerular images. This system includes preprocessing, glomerular structure segmentation, biomarker extraction, and glomerular and patient-level classification. For the deep learning methods, three different pre-trained CNNs are used to increase performance and decrease model overfitting. The deep learning approaches incorporated pre-processing, data augmentation, transfer learning, Grad-CAM for network visualization, and glomerular and patient-level classification. All methods are compared to the gold standard renal pathologist diagnosis of MCD, MN and TBMN.

### 2.3 Biomarker feature extraction (BFE) model

The biomarker feature extraction and machine learning model proposed in Basso et al. (5) was replicated in this work. Pre-processing steps are first employed to exclude small glomeruli, as well as color normalization to remove color variability using a modified version of Reinhard’s method (5). Three sub-glomerular structures were automatically segmented using a modified Naïve Bayes classifier: (1) luminal (space inside the Bowman’s capsule and the capillary lumen), (2) glomerular tuft (the glomerular basement membrane (GBM) and mesangial matrix), and (3) nuclei (5). A series of biomarkers are extracted from the sub-glomerular structures, which includes color, morphological, and microstructural texture features, forming a total of 233 biomarkers (5).

The concentration of color(s), related to stained microscopic anatomy, will be analyzed using the histogram of the color channels (of different objects), through the mean, variance, skewness, kurtosis, energy, and entropy. These features describe the amount of structures (stain) present in the glomerulus. From the sub-glomerular structures, morphological features will be extracted to quantify shape and object-based characteristics. Four groups of morphological features are included: containment features, shape features, interstructural distance features, and intrastructural distance features (5). Containment features measure the fraction of two areas. Shape features which include equivalent diameter and circularity will be used quantify the diameter of the glomerular structures and the roundness of the glomerulus. Interstructural distance features will assess distance between glomerular structures and are used to describe how structures interact with each other. Lastly, intrastructural distance features will be used to measure the thickness of a structure (e.g., glomerular tuft max thickness). The Euclidean distance transform was used to measure the thickness of each glomerular structure, yielding feature images that quantify spatial thickness. From these images, maximum, median, and total thickness features were extracted. Microstructural texture features were designed to measure spatial relationships between color or gray level pixels and describe glomerular microstructure tissue texture. Local and global texture-based biomarkers were evaluated using gray-level co-occurrence matrices (GLCM), color vector local binary patterns (LBP), and wavelet features (5). From Basso et al., Gini feature importance to select the features and Linear Discriminant Analysis (LDA) classifier combination was optimal (5) and are used for this analysis.

The dataset will be split into training, validation, and testing sets based on patients. Glomeruli are assigned disease labels based on the WSI-level label. Five-fold cross-validation will be used for classifier hyperparameter fine-tuning and all results are reported on the held-out testing set. The output of the LDA classifier is a probability for each disease, and the maximum probability will determine the predicted glomerular disease label.

### 2.4 Deep learning methods

A deep learning-based computer-aided system was applied to microscopy PAS images for MCD, MN, and TBMN glomerular classification. The following deep learning procedure included image pre-processing, data augmentation, transfer learning, Grad-CAM for network visualization, and glomerular classification. Models were trained on a computer with a NVIDIA GeForce 2080 SUPER GPU with 16 GB of RAM.

#### 2.4.1 Pre-processing

**Figure 3:**
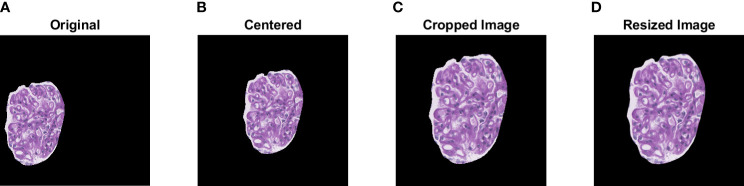
Visualization of the pre-processing steps prior to data augmentation. A sample glomerular ROI is shown in **(A)**. Centering, cropping, and resizing of the original ROI are shown in **(B–D)**.

The TGH dataset is first preprocessed prior to training and testing the deep learning models. Automatic glomerular size outlier detection was performed to remove small glomeruli from the analysis. Four glomeruli were found to be outliers reducing the TGH dataset from 375 to 371 glomeruli images. Next glomerular resizing needs to be completed, since deep learning methods accept predefined image sizes. To complete this, ROI re-centering, cropping, and then resizing is performed. To find the appropriate areas for resizing, a bounding box was computed for each centered glomerulus image and the largest bounding box width/height across the dataset was recorded. This max length was used as the max size to crop the images to keep it consistent over the entire dataset. Cropping the images to their minimum size allows for better resizing. Glomerular images were cropped from 1,500 x 1,500 pixels to 1,151 x 1,151 pixels in dimension. The InceptionV3 model which is one of the top performers on ImageNet has a maximum image size of 299 x 299 pixels, whereas VGG16 and VGG19 have 224x244 pixels. The cropped images are resized to these dimensions using bicubic interpolation and an example is shown in **Figure 3** below. Color normalization was also investigated using a modified version of Reinhard’s method which decreases color variability in WSIs (3, 4, 7).

**Figure 4:**
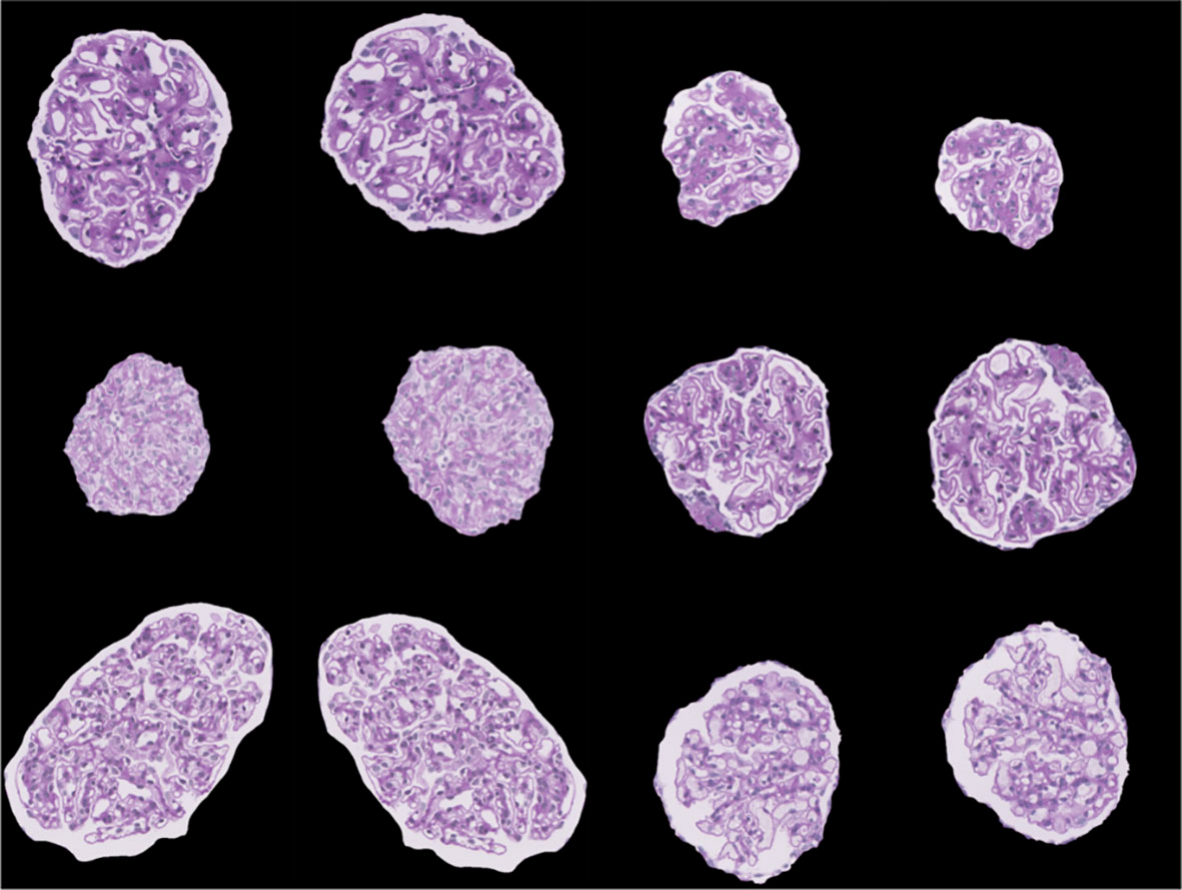
Sample images visualizing the result of data augmentation (rotation, width shift, height shift, zoom, horizontal flip, and vertical flip).

Once the images were preprocessed, data augmentation was utilized. Deep CNNs require large amounts of training data to help learn features from the data. Training an architecture using a small dataset may result in overfitting. Thus, to overcome these problems, different data augmentation techniques such as horizontal and vertical flip, rotation, scaling, and translation were used to increase the dataset size and help improve classification performance (8). The data augmentation parameters are shown in **Table S2**, and sample augmented images are shown in **Figure 4**.

#### 2.4.2 Transfer learning

A transfer learning approach was implemented based on three well-known deep CNNs to automatically initialize network weights for better generalization to the glomerular image diseases (9). Transfer learning is a method in which a network is pre-trained on a large dataset and used for a new problem (10). This technique is a popular approach in deep learning because it requires little data to train a deep CNN and has the potential to reduce the problem of overfitting (9). Although the pre-trained dataset is not the same as the source dataset, many low-level generic features such as edges, textures and contours extracted using the CNNs can be represented across datasets. This technique is valuable in medical imaging and digital pathology applications since expert annotations may not be available or data may be scarce for certain applications.

Three widely used pre-trained architectures were considered for glomerular classification: VGG16 (11), VGG19 (11), and InceptionV3 (12) since they have top performance in several challenges, are not extremely deep compared to other CNNs, and pre-trained weights are available. The VGG16 network has a total of 16 trainable layers, of which 13 layers are convolutions and 3 fully connected layers. Similarly, the VGG19 architecture has a total of 19 trainable layers, with 16 convolutional layers and 3 fully connected layers. Both VGGNets use an input image size of 224 x 224 x 3 and therefore resizing was performed. InceptionV3 was designed to resemble a fully connected deep neural network by utilizing sparsely connected modules. The InceptionV3 modules in turn help prevent overfitting and reduces the number of parameters needed which are key disadvantages when training a deep model on small amounts of data. Each InceptionV3 module is composed of small convolutional and pooling layers in parallel. For this architecture, an input image size of 299 x 299 x 3 was used. CNNs were pre-trained on a large collection of images from ImageNet (13).

For VGG16 and VGG19, a global average pooling (GAP) layer was added to replace the last maximum pooling layer. InceptionV3 already had a global average pooling layer and thus was not changed. GAP reduces the number of parameters needed to be trained to minimize overfitting (especially on smaller datasets), and can increase classification performance (14). The number of parameters of VGG16 and VGG19 with and without the added GAP layers can be found in **Table S3**. GAP layers reduced the number of parameters of the VGG networks by approximately 150 million while maintaining the same network depths. With GAP layers added, VGG16/VGG19 are more comparable in terms of number of trainable parameters compared to the InceptionV3 network.

**Figure 5:**
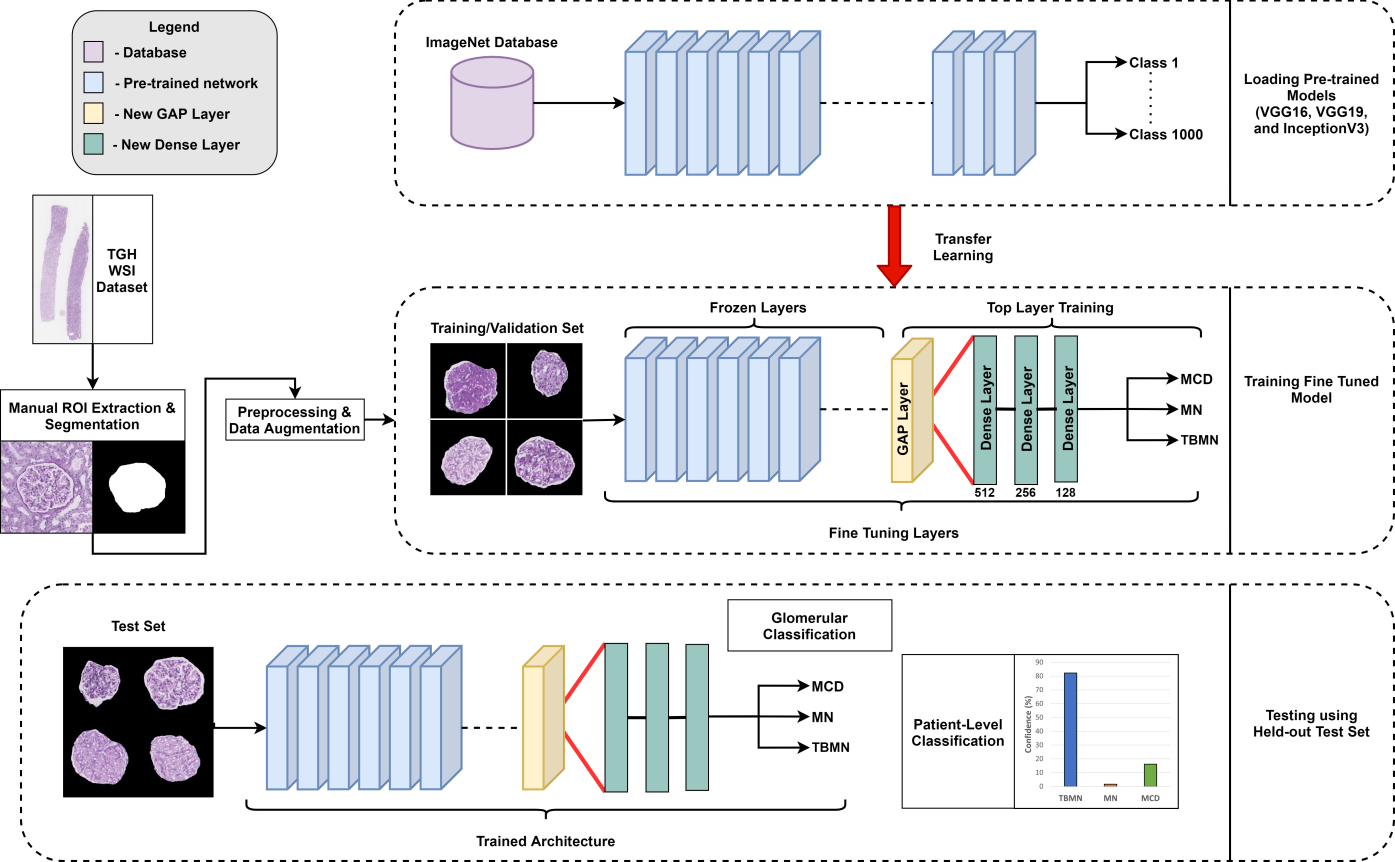
Overview of experimental design. Three different pre-trained networks (VGG16, VGG19, and InceptionV3) were used in a fine-tuning approach.

Three new trainable fully connected layers were then added to all three models and an output vector of length three was added to match the number of labels (three) for the candidate glomerular diseases. A 50% dropout was added between the fully connected layers to prevent overfitting. The weights from all three pre-trained architectures are initially frozen and the new final layers are trained on the TGH dataset for each of the architectures. After training the new final layers on the TGH dataset, additional fine-tuning was completed, whereby all model weights were unfrozen and training was completed on the TGH dataset with a small learning rate. Fine-tuning was performed to help adapt the pre-trained features to the glomerular image dataset. The output of the network is a prediction probability (softmax) for each glomerular disease, and the maximum probability is used to predict the glomerular level label. Hyperparameters for the models are show in **Table 1**. Modifications to the architectures, implementation, and fine-tuning approach for VGG16, VGG19, and InceptionV3 is demonstrated in **Figure 5**.

**Table 1:** Model hyperparameters.

Parameter		Value
Optimizer		Stochastic Gradient Descent (SGD)
Loss		Categorical Cross-Entropy
Momentum		0
Learning Rate	
* Top Layer Training*		0.01
* Fine Tuning*		0.001
Dropout		0.5
Mini Batch Size		32
Maximum Epochs		100

#### 2.4.3 Grad-CAM visualization

Deep learning models may lend to a lack of interpretability (15, 16) and methods such as Gradient-weighted Class Activation Mapping (Grad-CAM) can add transparency by visualizing important features in the image used to make the decision (17). Grad-CAM uses gradients of the predicted disease class and creates a heat-map indicating regions of importance. Grad-CAM was implemented for each of the trained networks and gradients were taken from the output predicted class as input into the last convolutional layer.

### 2.5 Patient-level classification scheme

For all machine learning methods (LDA and CNNs), the output is a probability distribution that specifies the probability the glomerulus image is from a subject with MC, TBMN or MN. The maximum probability value is used to predict the disease label for glomerulus-level classification. To automatically predict the disease on a patient level using the held-out test set, each glomerulus is first classified from the subject’s renal biopsy. The top four glomeruli predictions with the highest probability are averaged to estimate WSI disease predication confidence and diagnosis. This method approximates human assessment as a pathologist may make a diagnosis from a few glomeruli that best exemplify a disease.

### 2.6 Validation metrics

Each subject has a WSI-level label of MCD, MN, or TBMN derived from pathologist (R.J.) assessment. These labels were compared to the classifier predictions. Some of the metrics to evaluate the model’s classification performance include accuracy, precision, recall, and F1-score. Accuracy measures the fraction of correct predictions over the total number of predictions. Where each true positive (TP), true negative (TN), false positive (FP), and false negative (FN) are found comparing true and predicted labels.


$$ACC=\frac{TP\;+\;TN}{TP+\;FP+\;TN\;+\;FN}$$

F1-score was also analyzed, which is a combination of both precision and recall and gives an overall accuracy score. A high F1-score indicates the classifier is predicting with high precision and recall.


$$F_{1}\;=\frac{2∙\;Precision\;∙\;Recall}{Precision\;+\;Recall}$$

## 3 Results

**Table 2** describes the experimental dataset used for the biomarker feature extraction and deep learning models, which was split by patient into 67% training/validation (*n* = 30 WSIs, *n* = 250 glomeruli) and 33% testing (*n* = 15 WSIs*, n* = 121 glomeruli). For the deep learning models, data augmentation previously described was used to increase the sample sizes in the training/validation set, with equal numbers between the diseases to prevent class imbalance.

**Table 2:** Data configuration for glomerular and patient-level classification.

	TGH Dataset		Training/Validation			Testing	
	Patients	Glomeruli	Patients	Glomeruli (BFE)	Glomeruli (DL)	Patients	Glomeruli
**MCD**	15	103	10	66	200	5	37
**MN**	15	148	9	101	200	6	45
**TBMN**	15	124	11	83	200	4	39
Total	45	375	30	250	600	15	121

The TGH dataset was composed of minimal change disease (MCD), membranous nephropathy (MN), and thin-basement membrane nephropathy (TBMN) WSIs. This dataset was split into training/validation and testing on a per patient basis. Glomeruli and patient-level classification was trained/validated and tested using the following configuration.

**Figure 6:**
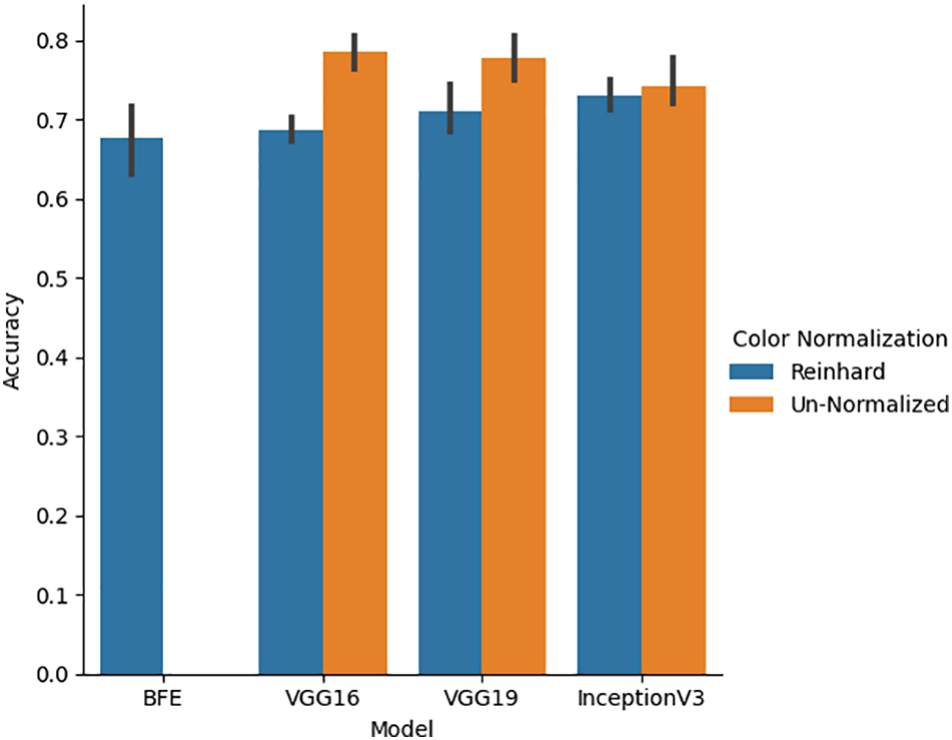
Five-fold cross-validation accuracy performance for BFE and deep learning models performed on validation set. A comparison between un-normalized and Reinhard color normalized images was additionally performed across all deep learning models.

Five-fold cross validation was performed on the validation set for all classifiers for both un-normalized and Reinhard color normalized images seen in **Figure 6**. Color normalization using Reinhard’s method which normalized the mean and standard deviation of each channel of an image to a target image was performed to facilitate color consistency throughout the dataset. On normalized data, the BFE model using Gini feature importance and Linear Discriminant Analysis obtained a glomerular cross-validation accuracy of 67.6 ± 8.9%. For the deep learning models, un-normalized images had higher cross-validation performance compared to Reinhard normalized images. The VGG16 model for un-normalized images had the highest cross-validation accuracy of 78.5 ± 4.6% and therefore generalized to the validation data the best. As shown by **Figure 6**, this color transformation reduced the deep learning model’s ability to differentiate between the diseases, thus only un-normalized images are used for the deep learning models.

The BFE and deep learning models for un-normalized data were applied to the glomerular unseen held-out testing set over all folds. Accuracy, precision, recall, and F1-score glomerular and patient-level classification results for each model are shown in **Table 3**.

**Table 3:** Glomerular five-fold cross validation and testing glomerular and patient-level classification for all models on the held-out test set.

Classification Metrics	BFE		VGG16		VGG19		InceptionV3	
Validation Accuracy	67.6 ± 8.9%		78.5 ± 4.6%		77.8 ± 6.5%		74.2 ± 6.8%	
Testing	*Glomerular*	*Patient-Level*	*Glomerular*	*Patient-Level*	*Glomerular*	*Patient-Level*	*Glomerular*	*Patient-Level*
Accuracy	76.86%	86.67%	71.90%	66.67%	63.64%	60.00%	64.46%	73.33%
Precision	76.55%	86.90%	71.59%	63.89%	64.92%	68.89%	62.39%	70.79%
Recall	76.40%	85.00%	70.43%	61.67%	61.58%	56.67%	62.90%	71.67%
F1-score	76.47%	85.94%	71.01%	62.76%	63.21%	62.19%	62.64%	71.23%

**Figure 7:**
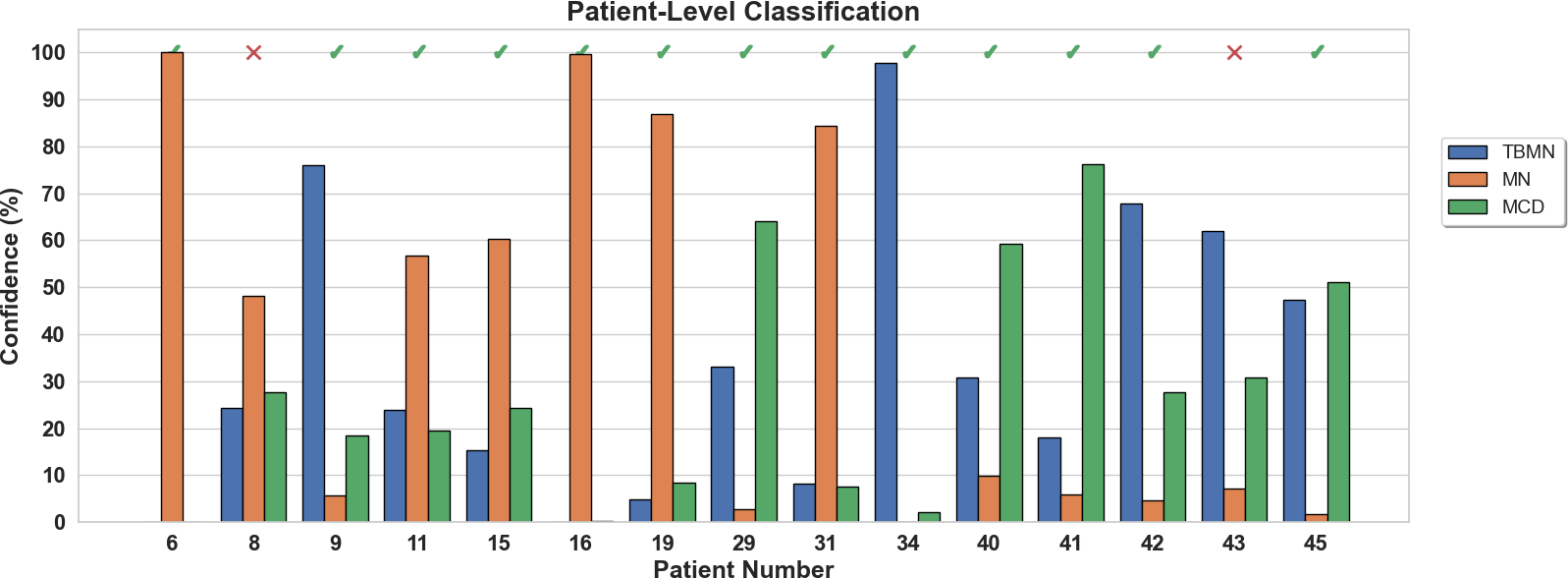
Patient-level confidence results per testing patient are shown for the BFE model. Each patient was predicted with a certain confidence corresponding to TBMN, MN, and MCD. Above the confidence bars is indicated whether the patient was predicted correctly with a checkmark and incorrectly predicted with an X.

The BFE model obtained a testing accuracy of 76.86% for glomerular-level classification. Glomerular classification accuracy was high for MN (84%), and lower for MCD (73%) and TBMN (72%). Patient-level classification was determined by the top four glomeruli on the held-out testing set which resulted in an accuracy of 86.76%. The confidence rating (average probability over the top 4 glomeruli) for each held-out subject is shown in **Figure 7**. As seen in **Figure 7**, the BFE method correctly predicted all patients except for 8 and 43, with an average subject-level confidence of 73 ± 17% for the predicted class. The correct disease labels were TBMN for subject 8 and MCD for subject 43, which were predicted to be MN and TBMN, respectively. Notice the confidence level for TBMN and MCD is similar over many patients, while correctly classifying MN with very high confidence.

**Figure 8:**
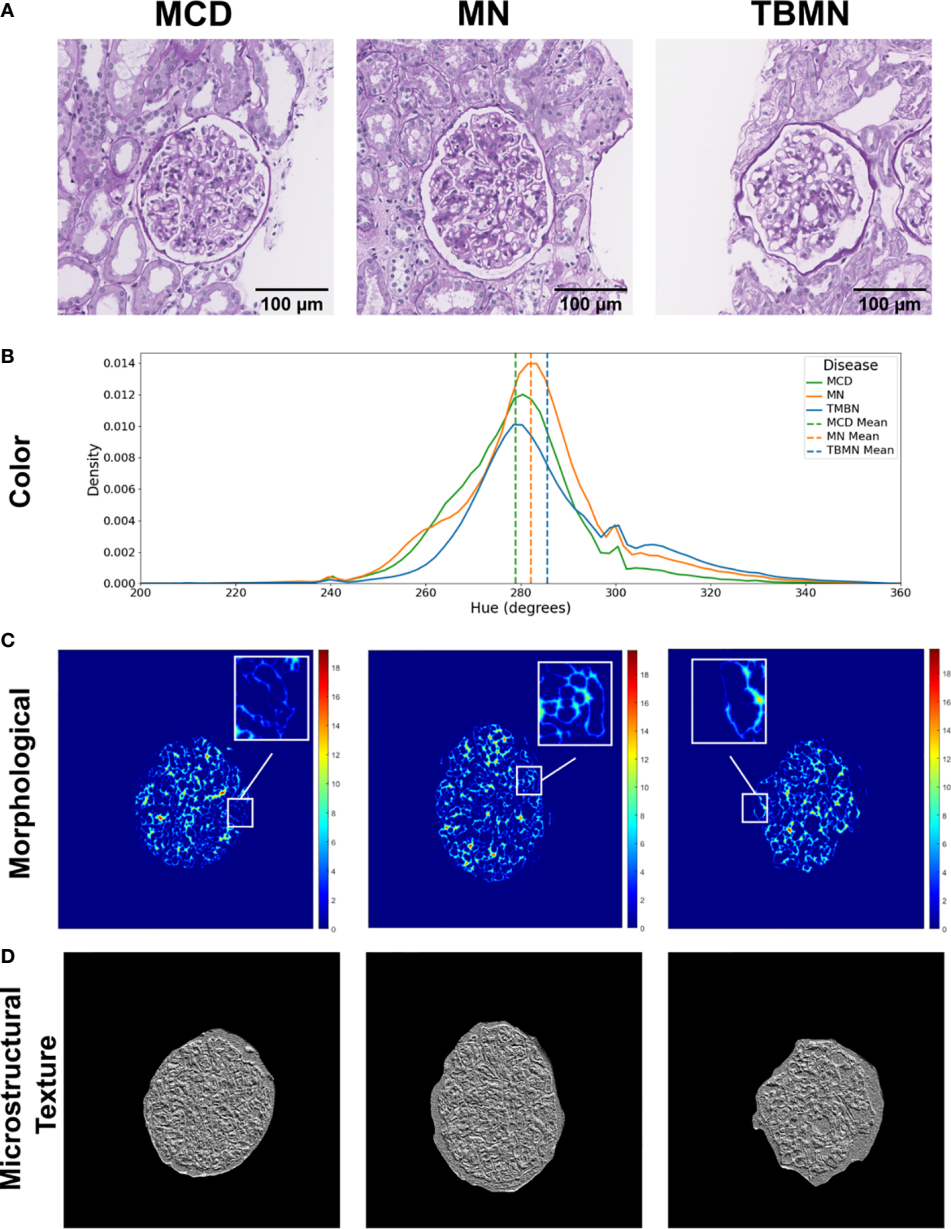
Visual representation of biomarker features extracted. **(A)** Columns represent sample minimal change disease (MCD), membranous nephropathy (MN), and thin-basement membrane nephropathy (TBMN) diseases. **(B)** Color features: displays the hue histogram for the following three sample images, with their corresponding mean values. **(C)** Morphological features: glomerular tuft intrastructural distance feature maps for each corresponding sample image. Thicker structures are represented as red or orange in color, while thinner structures are green and blue in color. **(D)** Microstructural texture features: texture maps for sample glomeruli using color vector LBP. Scale bars, 100 µm.

**Figure 8** illustrates the three biomarker groups from which 233 features were extracted as part of the BFE model. The biomarkers obtained were designed to be interpretable and describe the underlying pathology. **S**ample glomeruli are shown in **Figure 8A**. First is the hue color histogram in **Figure 8B**, which shows the TBMN glomerulus to have the highest mean hue indicating increased ‘luminal structure’. **Figure 8C** shows the intrastructural distance feature and zoomed-in regions show the GBM in yellow for MN indicating thickening, while the GBM is dark blue for TBMN, compatible with thinning. Lastly, color vector LBP texture maps, which quantify ultrastructural spatial relationships between pixels, are illustrated in **Figure 8D**. MCD and TBMN glomeruli show finer texture than MN, which is likely from increased ‘glomerular tuft’ in MN.

**Figure 9:**
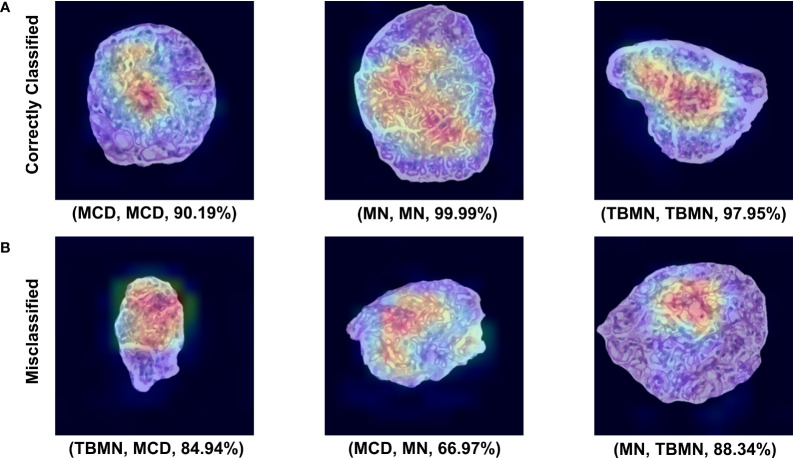
VGG16 Grad-CAM heatmaps for the top correctly predicted and misclassified glomeruli with respect to disease. **(A)** Sample glomeruli that were predicted correctly. **(B)** Sample glomeruli that were misclassified. The notation below each image represents (predicted disease, actual disease, probability). Areas of importance are colored red or orange, while less important regions are colored blue.

All three deep learning models (on unnormalized data) had lower glomerular-level testing classification accuracy. All three models were found to slightly overfit the training data as seen by the loss graphs in **Figures S1-3**. Although steps such as pretraining, data augmentation, model fine-tuning, and added layers were completed to mitigate model overfitting, deeper networks perform best in a large data scenario. The VGG16 method performed the best with a glomerular testing accuracy of, 71.90%. Much like the BFE model, MN was found to have the highest classification accuracy (96%) but substantially lower performance for MCD (57%) and TBMN (59%) was found as shown in **Table 4**. MCD and TBMN are misclassified as one or another, in greater proportion compared to MN. Grad-CAM was used to visualize the important features in the glomerular images to add explainability. The correctly classified and misclassified glomeruli (with highest probability) in each disease are shown in **Figure 9**. Areas of red and orange indicate regions in the images that were deemed important for making a prediction, while less important regions are colored blue. From these images, areas of importance pointed to glomerular tissues and nuclei structures. The correctly classified MN image highlighted the thicker glomerular tuft structure as important, which potentially could have been a feature during classification. Similarly, the misclassified TBMN image was predicted as MN possible due to the detection of thicker membrane which was highlighted. Although areas of interest are highlighted, in some of the misclassified glomeruli, there was outside regions included, or just a small region that was deemed important.

**Table 4:** Confusion matrix containing glomerular-level classification of MCD, MN, and TBMN using the fine-tuned VGG16 network.

		Target Class		
		MCD	MN	TBMN
**Output Class**	**MCD**	21 (57%)	2 (4%)	7 (18%)
	**MN**	7 (19%)	43 (96%)	9 (23%)
	**TBMN**	9 (24%)	0 (0%)	23 (59%)

For patient-level classification, although VGG16 had the highest glomerular-level accuracies, VGG16 had the second lowest patient-level classification accuracy. Recall that predicting slide-level disease is based on the top four glomeruli with the highest probabilities. Since the classifier had difficulties differentiating TBMN and MCD, VGG16 resulted in a lower patient-level performance as there was disagreement in the labels for the top four glomeruli.

The InceptionV3 network, while having the lowest glomerular classification accuracy of 64.46%, was found to have the highest patient-level accuracy of 73.33%. Upon further inspection (**Table 5** confusion matrix), this model was found to classify 100% of the MN patients correctly, while 75% of the TBMN and 40% of the MCD patients were predicted correctly. In this case, the classifier is robustly representing MN patients, as well as patients with TBMN. MCD was still difficult to differentiate.

**Table 5:** Confusion matrix containing patient-level classifications of MCD, MN, and TBMN using the fine-tuned InceptionV3 network.

		Target Class		
		MCD	MN	TBMN
**Output Class**	**MCD**	2 (40%)	0 (0%)	1 (25%)
	**MN**	1 (20%)	6 (100%)	0 (0%)
	**TBMN**	2 (40%)	0 (0%)	3 (75%)

**Figure 10:**
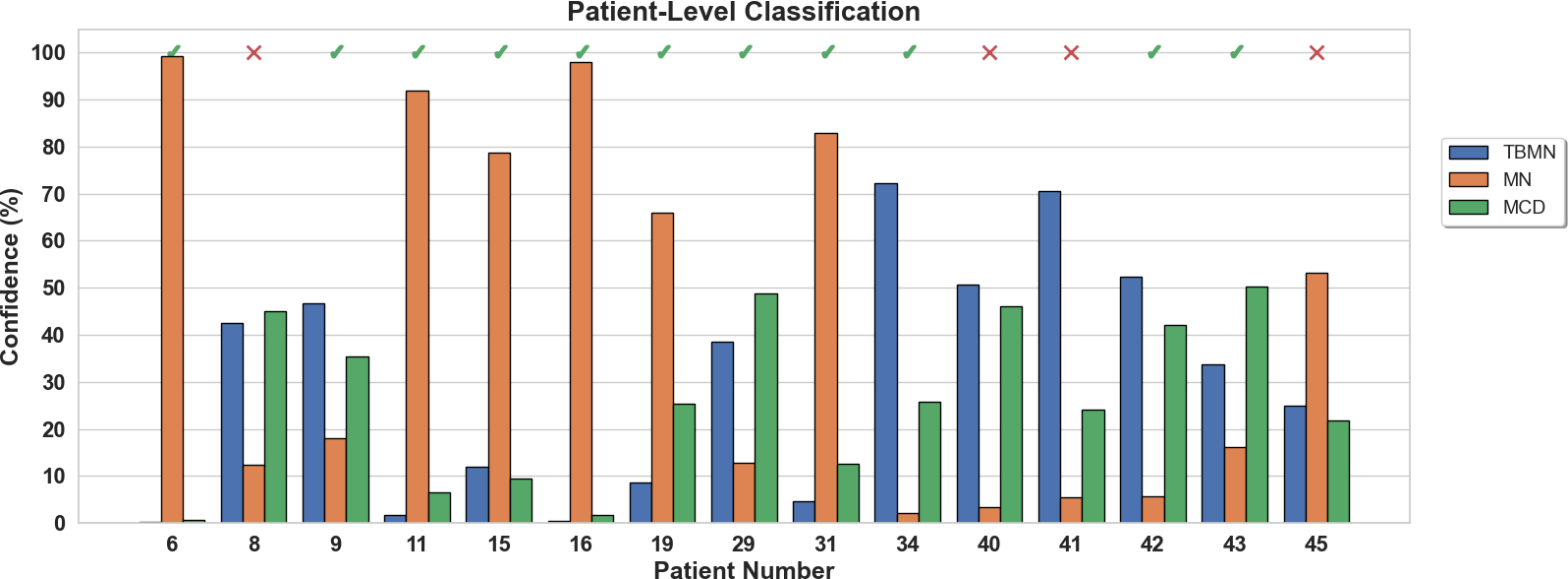
Patient-level confidence results per testing patient are shown for the InceptionV3 deep learning model. Each patient was predicted with a certain confidence corresponding to TBMN, MN, and MCD. Above the confidence bars is indicated whether the patient was predicted correctly with a checkmark and incorrectly predicted with an X.

The confidence rating (average probability over the top 4 glomeruli) for each held-out subject is shown in **Figure 10** for the InceptionV3 network. As seen in **Figure 10**, 4 patients (8, 40, 41, and 45) were misclassified with an average confidence of 67 ± 19%. The labels for subjects 8, 40, 41 and 45 were TBMN, MCD, MCD and MCD, which were respectively predicted to be MCD, TBMN, TBMN and MN. Interestingly, similar to the BFE model TBM and MCD had similar probabilities, and MN was predicted with high confidence.

## 4 Discussion

Our study is unique in using PAS-stained renal biopsy WSIs and machine learning to diagnose glomerular disease. To diagnose glomerular disease clinically, LM, IF, and EM are required which is laborious, costly, and can have delays in turn-around time. The use of computer-aided diagnostics can present a significant gain, particularly if EM and/or IF is not available, but also potentially as a screening tool, opening up pathologist time and healthcare resources. PAS WSIs also allow for remote diagnosis *via* cloud-based technology. In this work, we designed and implemented several machine learning algorithms that use explainable biomarkers and traditional machine learning, as well as three deep learning systems to automatically classify between MCD, MN, and TBMN diseases on a glomerular and patient-level. This proof-of-concept study was an investigation into two renal disease tools and describe how these methods can be designed and validated for renal pathology images. The results of the various models investigated across three glomerular diseases using PAS images only has shown promise.

Biomarker feature extraction and deep-learning computer aided diagnostic systems were applied to the PAS light microscopy experimental dataset. The BFE model resulted in a glomerular five-fold cross-validation accuracy of 67.6 ± 8.9% and testing accuracy of 76.86%. Amongst the three CNNs, the VGG16 network was the top deep-learning model for glomerular classification with a cross-validation of 78.5 ± 4.6% and a testing accuracy of 71.90%. Patient-level classification had slightly higher average performance for the BFE compared to all the CNN methods, with two misclassified patients, vs. four for the InceptionV3 model. Color normalization was also tested for the deep learning models and it was found to reduce classification performance. In previous works, color normalization of digital pathology images resulted in similar nuclei segmentation performance based on un-normalized datasets (18) but was noted that segmentation can be negatively impacted by color normalization if a reduction of contrast was found, which may be the case in this work as the dataset was from the same center.

The biomarkers (features) from the BFE model (5, 19) were designed to be related to underlying pathology (tissue micro- and macro- structure) to give the user confidence in the system and to further understand disease by identifying subvisual features. The selected features suggested that MCD had larger nuclei, possibly related to podocyte hypertrophy, MN had glomerular tuft thickening, and TBMN had glomerular tuft thinning. Microstructural texture features described TBMN and MCD to be heterogeneous, possibly from variable nuclei and luminal structures, while MN was homogeneous, likely related to glomerular tuft thickening. Overall, this traditional machine learning approach performed moderately on a small dataset. At the patient-level, all MN cases were classified correctly, while one MCD subject and one TBMN subject were misclassified.

VGG16 was the best of the CNNs for glomerular classification, which classified MN with near perfect classification accuracy, whereas TBMN and MCD performed poorly. This is likely due to similar features in these diseases, including thicknesses of the glomerular basement membrane. To try and tease out the subtle differences between these diseases as seen on EM, we used pre-trained networks and fine-tuning since the labeled dataset in this work was small, which is not uncommon in medical imaging and further exacerbated in the renal pathology space. In the future, we will consider incorporating a larger dataset, which would likely improve the deep learning model performance. Another consideration is the pre-trained networks were developed using natural images, which are different than pathology images, and that may have affected performance. Perhaps the fundamental features between the datasets are too different, causing suboptimal performance. In the future, we will consider pre-trained models developed on pathology images, such as (20). We can then test if using a pathology pretrained network and the small dataset for fine-tuning, improves performance.

InceptionV3 had lower glomerular classification accuracy compared to the VGG architectures, but highest patient-level accuracy where only 4 out of 15 cases were misclassified. This likely indicates more homogeneity in the InceptionV3 classification predictions for the top four glomeruli. Glomeruli were correctly classified 100% of the time for MN disease, followed by TBMN (75%) and then MCD (40%). Higher misclassifications were made between TBMN and MCD, indicating, as supported by results from all the other methods, that differentiating between MCD and TBMN on PAS stained images is more difficult. Thicker membranes, and other features, likely make MN easier to differentiate. In the future, we may consider a binary classification problem between MCD and TBMN to further find differences in images for the PAS-stained renal biopsies.

With regards to technical aspects, developing traditional machine learning and deep learning each require a set of domain expertise. Hand-crafted feature design is needed to extract features (inputted to the machine learning tool), which is completed using signals and systems theories; but designing algorithms that detect the phenomena of interest can be difficult. In contrast, deep learning systems use data to learn these features automatically, and there are many architectures available. Learning to modify and fine-tune pretrained architectures for the dataset and task requires specific skill sets as well. However, a challenge with machine learning is that large datasets are usually required for the best performance and generalization – and deep learning methods require the most data to learn highly non-linear and complex relationships (through many parameters) which is important for describing disease. Currently, there are very limited datasets available for renal pathology, and resultantly very few automated methods for renal pathology as well. As described by a recent review of our BFE method (19), that despite the limited work in the machine learning and renal pathology space to date, especially for deep learning approaches (19, 20), our work demonstrates that machine learning is a viable and promising technology for this field. In the future, more labeled renal pathology images and methods are needed to continue to advance this space. Perhaps self-supervised learning methods can be utilized to exploit the massive amounts of unlabeled datasets (21). While developing larger models, with larger datasets results in higher performance gains, there is a trade-off with these models, including the amount of CO2 emissions generated by computers used to create the tools. Training one particular type of deep learning model (big Transformer model with neural architecture search) was equivalent to the entire lifetime CO2 emission of five cars (21). However, as shown in this work, the deepest models may not be necessary.

Soft factors related to perception (interpretability, explainability, trust) and impact (society, clinical, economic, and environmental) may also influence adoption (22). Likely important are limitations in the interpretability or transparency of the model (22), although a recent survey shows that providing the pathologist with information regarding the underlying model’s opaqueness did not affect pathologist decisions (23). There has been much discussion in the AI community about the limitation in interpretability and transparency of deep learning systems due to the “black box” phenomena (15, 16). At the same time, an argument has been made in favor of performance over explanation if the system is rigorously tested and validated (22). To increase trust, both models integrated a confidence rating for patient-level classification and cases with low confidence can be flagged for secondary review through traditional IF/EM. The added benefit of an associated confidence measure helps with borderline or questionable predictions for further review. Both image analysis and deep learning (if random initialization is the same for each run) can be highly reproducible, and hence more objective than human-based analysis. The BFE model is interpretable since biomarkers are related to underlying pathology (podocyte injury/loss and glomerular basement membrane thickening). Additionally, GRAD-CAM was used to highlight important features in the images that were used to make the predictions for the deep learning-based methods. Interpretability methods are quickly evolving in the AI field, and new methods to understand more about the decision of the classifier are on the horizon.

In terms of practical implementation and clinical workflow augmentation, deep learning may suffer from generalization problems, which arises when testing on data from new labs (24). This can be an issue for translation since most tools are developed using a single dataset (or similar) which may not operate as well or expected on unseen datasets from different labs. Methods such as augmentation and color normalization have been used to try to reduce this variability (7), and more recently, domain adaptation methods (25) are being explored to overcome this challenge. Feature extraction methods, on the other hand, depend on normalization to create consistent colors across scanners and datasets.

In the future, to improve this work, several modifications can be made. For the BFE method, additional features can be explored, such as ones dedicated to shape, texture and object sizes or thickness. These characteristics are related to the features that pathologists consider when using secondary modalities such as EM. Additionally, more advanced multi-resolution features (Gaussian scale space, Gabor) can be investigated. To improve the deep learning methods, in future work, the first consideration will be increasing the dataset size and experimenting with more recent models such as Resnets or Densenets. Additionally, as more pre-trained networks emerge that are domain specific, it would be interesting to compare the classification results based on natural images to models created using pathology images. As new interpretability maps emerge, these will also be experimented with as well.

The major limitation of this study is the small dataset which likely led to less-than-optimal CNN performance. Deep learning models require large amounts of data to learn descriptive features and to prevent overfitting. The inclusion of only three glomerular diseases could also be seen as a limitation, given the wide range of glomerular diseases that pathologists encounter. However, most previous works only focused on two labels, so this work is the first automated work for three kidney diseases. In the future, we will extend these frameworks to additional glomerular diseases and lesions, and will report on our findings.

## Data availability statement

The datasets presented in this article are not readily available because Data cannot be shared due to privacy and confidentiality of the patient. Requests to access the datasets should be directed to mnbasso@ryerson.ca.

## Ethics statement

The studies involving human participants were reviewed and approved by Toronto General Hospital Research Board Approval (19-5268). The patients/participants provided their written informed consent to participate in this study.

## Author contributions

MNB performed the formal analysis, investigation, methodology, validation, and writing of the original draft. MB and RJ curated the data and acquired funding. AK was responsible for the supervision of the study and acquired funding. All authors including JM conceptualized the study, validated the results, and reviewed and edited the manuscript.

## Funding

This work was supported by Faculty of Engineering & Architectural Science, Ryerson University (AK), Alport Syndrome Foundation, and Government of Canada, Canadian Institutes of Health Research (CIHR).

## Acknowledgments

We would like to acknowledge Ryerson University’s Dean’s Research Fund (DRF) program for funding this research. Additionally, we thank KRESCENT, Alport Syndrome Foundation, McLaughlin Centre- University of Toronto, NephCure Kidney International-Neptune, Can-SOLVE CKD Network, the Toronto General Hospital Foundation and CIHR for their support.

## Conflict of interest

The authors declare that the research was conducted in the absence of any commercial or financial relationships that could be construed as a potential conflict of interest.

## Publisher’s note

All claims expressed in this article are solely those of the authors and do not necessarily represent those of their affiliated organizations, or those of the publisher, the editors and the reviewers. Any product that may be evaluated in this article, or claim that may be made by its manufacturer, is not guaranteed or endorsed by the publisher.
