# Supplementary material for: Machine learning in renal pathology
Source: Front Nephrol. 2022 Nov 29;2:1007002. doi: 10.3389/fneph.2022.1007002 (PMC10479587; doi:10.3389/fneph.2022.1007002)
Supplement: Supplementary file 1 [file DataSheet_1.pdf]

Supplemental Material

Clinical Information

The dataset was obtained from Toronto General Hospital (TGH) in Toronto, Ontario, Canada and has institutional research board approval. This dataset consists of WSIs of renal biopsies from n = 45 different patients with n = 15 WSIs per disease (MCD, TBMN, and MN), derived from pathologist (R.J.) assessment using LM, IF, and EM. **Table S1** details additional clinical information such as age, sex, and disease specific information.

Table S1. Additional clinical information from the TGH dataset.

Collective Statistics	Value	MCD Statistics	Value	MN Statistics	Value	TBMN Statistics	Value
Number of Patients	45	Number of Patients	15	Number of Patients	15	Number of Patients	15
Number of Glomeruli	375	Number of Glomeruli	103	Number of Glomeruli	148	Number of Glomeruli	124
Age (years)		Age (years)		Age (years)		Age (years)	
Mean (SD)	48.1 (17.3)	Mean (SD)	44 (21.2)	Mean (SD)	54.5 (16.1)	Mean (SD)	45.8 (11.2)
Median (Range)	49 (19-95)	Median (Range)	41 (19-95)	Median (Range)	53 (26-81)	Median (Range)	43 (31-64)
Sex		Sex		Sex		Sex	
Male (%)	20 (44.4%)	Male (%)	10 (66.7%)	Male (%)	9 (60%)	Male (%)	1 (6.7%)
Female (%)	25 (55.6%)	Female (%)	5 (33.3%)	Female (%)	6 (40%)	Female (%)	14 (93.3%)
		Foot Podocyte Effacement		PLA2R		Genetic Testing	None
		Mean (SD)	92.7 (9.3)	Negative (%)	3 (20%)		
		Median (Range)	100 (70-100)	Positive (%)	9 (60%)		
				Not Determined (%)	3 (20%)		

Deep Learning Pre-processing

Table S2. Data augmentation techniques performed.

Methods	Parameters
Rotate	-90, 90 degrees
Width Shift	50 pixels
Height Shift	50 pixels
Zoom	50% larger
Horizontal Flip	25% probability
Vertical Flip	25% probability

Table S3. Comparing number of deep learning network parameters (in million). Bolded values indicate a decrease in number of parameters when using global average pooling (GAP) layer.

Network	Number of Parameters (in million)
VGG16	165.73
VGG16-GAP	**14.88**
VGG19	171.05
VGG19-GAP	**20.19**
InceptionV3	22.36

Deep Learning Training/Validation Curves

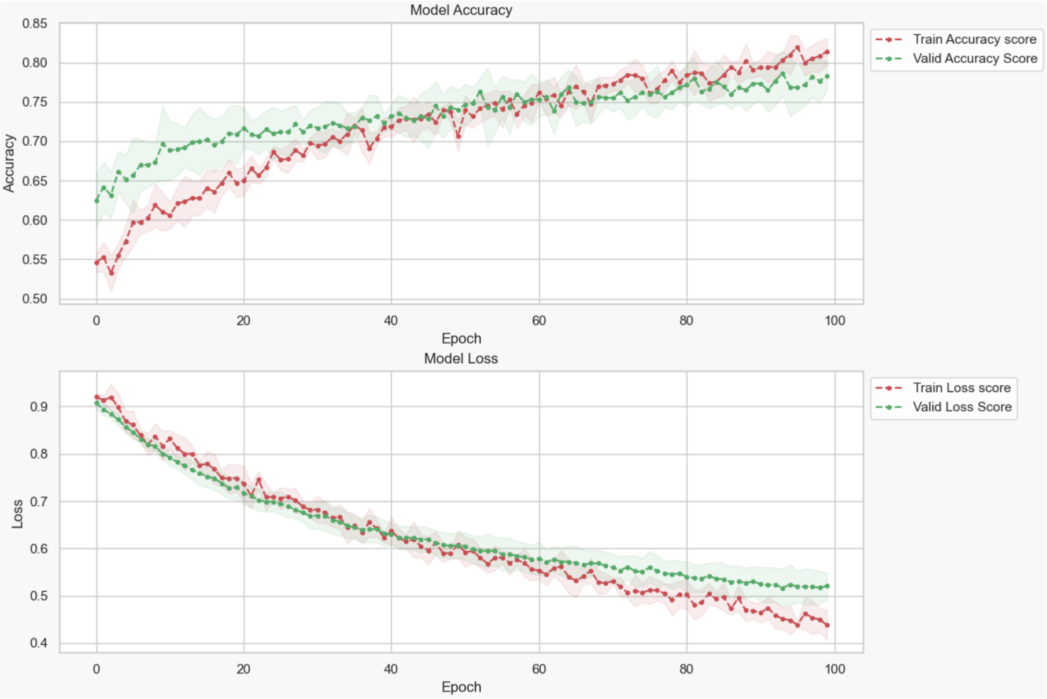

Figure S1. VGG16 five-fold training and cross-validation curves. Model accuracy and loss are plotted against the number of epochs.

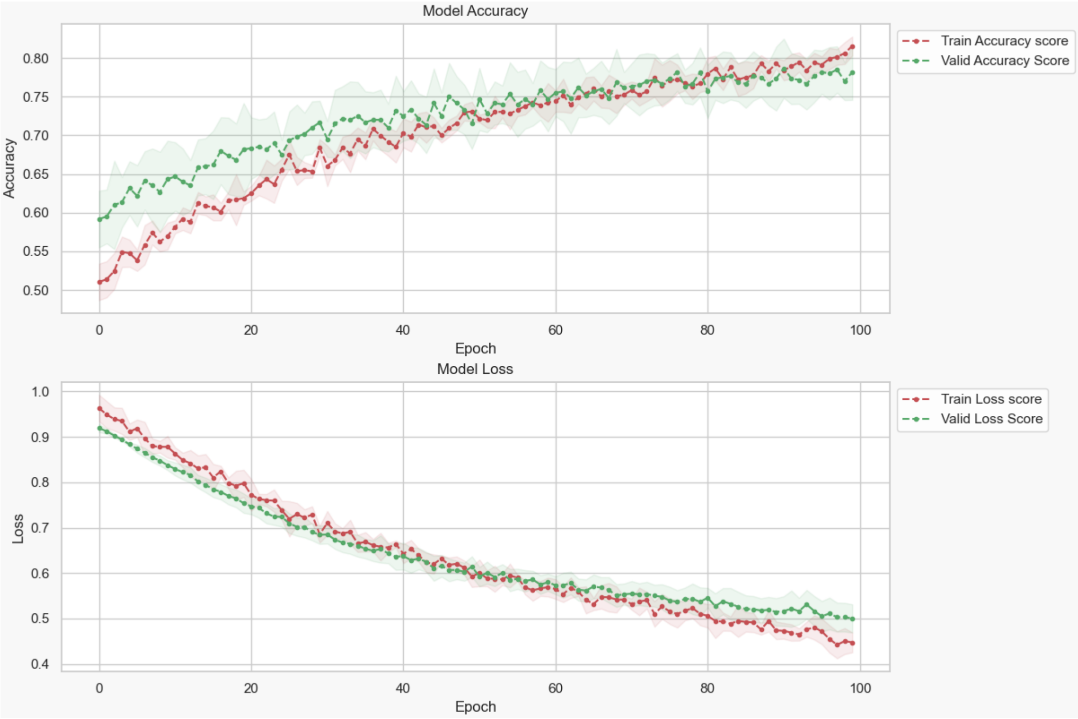

Figure S2. VGG19 five-fold training and cross-validation curves. Model accuracy and loss are plotted against the number of epochs.

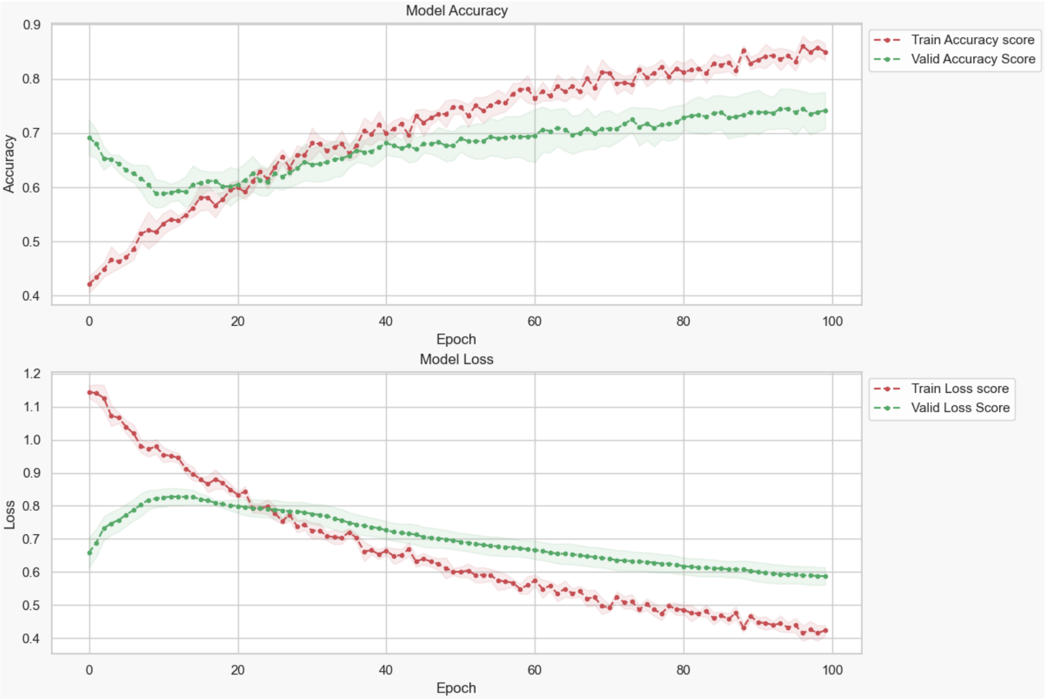

Figure S3. InceptionV3 five-fold training and cross-validation curves. Model accuracy and loss are plotted against the number of epochs.
